# Scapular deformity in obstetric brachial plexus palsy and the Hueter-Volkmann law; a retrospective study

**DOI:** 10.1186/1471-2474-14-107

**Published:** 2013-03-22

**Authors:** Valerie M van Gelein Vitringa, Barend J van Royen, Johannes A van der Sluijs

**Affiliations:** 1 Department of orthopaedic surgery, Spaarne Ziekenhuis, Spaarnepoort 1, 2134 TM Hoofddorp, The Netherlands; 2 Head of department of orthopaedic surgery, VU medical center, De Boelelaan 1117, 1007 MB Amsterdam, The Netherlands; 3 Department of orthopaedic surgery, VU medical center, De Boelelaan 1117, PO box 7057, 1007 MB Amsterdam, The Netherlands

**Keywords:** Hueter-Volkmann law, Obstetric brachial plexus lesion, Scapular bone growth, Shoulder deformity

## Abstract

**Background:**

The Hueter-Volkmann law describes growth principles around joints and joint deformation. It states that decreased stress leads to increased growth and that excessive stress leads to growth retardation. Aim of this study was to test the possible results of this principle by measuring the effect of dorsal humeral head subluxation on scapular growth in children with Obstetrical Brachial Plexus Lesions (OBPL). According to the Hueter-Volkmann law, subluxation should result in decrease of growth of the dorsal length of the scapula (by increased dorsal pressure) and increase of the ventral length (decreased pressure).

**Methods:**

58 children (mean age 20 months, range 1-88 month) with unilateral OBPL and good quality MRI of both shoulders were included. On MRI, humeral head subluxation, joint deformation, and ventral and dorsal scapular lengths were measured. Data were expressed as a ratio of the normal side.

**Results:**

In affected scapulas both ventral and dorsal side were smaller compared to the normal side. Reduction of growth on the affected side was more marked on the dorsal side than on the ventral side (6.5 mm respectively 4.5 mm, p < 0.001). The balance of growth reduction as expressed by the ratio of ventral and dorsal length was strongly related to subluxation (R^2^ = 0.33, p < 0.001) and age (R^2^ = 0.19, p < 0.001).

**Conclusions:**

The Hueter-Volkmann law is incompletely active in subluxated shoulders in OBPL. Dorsal subluxation indeed leads to decrease of growth of the dorsal length of the scapula. However, decreased stress did not lead to increased growth of the ventral length of the scapula.

## Background

One of the principles that appears to partially govern bone growth is the Hueter-Volkmann “law“. More a principle than a law, it was formulated to describe growth principles around immature joints and is paraphrased stating that excessive pressure to a part of the joint leads to local growth retardation and reduced pressure to a part of the joint leads to local growth acceleration.

Although in its paraphrased form it describes the relation between pressure and bone growth, the concept of pressure was originally less stressed by Hueter. In the original 1862 article Hueter stresses more the influence of movement and joint position on joint form [1]. Sentences giving his ideas in this 36 pages article written in the extended style of the epoch are (authors translation): (p485) “In this flexion position only the posterior parts of the tibia are in contact with the joint surfaces of the femoral condyles while the anterior parts of the tibia are not in contact with the femur and thereby are free of the pressure that is resting on the posterior parts. Because of this pressure difference the anterior part develops a relative larger length growth of the diaphyses than the posterior part. while the epiphyseal cartilage … shows no difference in height between anterior and posterior sections …”. In another part of his article: (p502) “In what I said describe above, I have tried to show the influence of movements on the various surfaces of the knee joint and on their form and remodelling”. Hueter stated more the influence of movements and position, but indirectly explains it in terms of pressure.

Originally formulated by Hueter, Volkmann’s name was later added to this growth principle, by his statements on the effect of tension and compression on the epiphyseal plate, coincidentally in the same year 1862 [2,3]. The Hueter-Volkmann law was later on generalised beyond joint growth and extended to describe growth and remodelling in long bones [4]. Since a rigid application of the principle, that excessive pressure leads to growth arrest would preclude all remodelling, it was adapted by suggesting that an increase in stress would first lead to increased growth and excessive stress to retardation [5-8].

The Hueter-Volkmann law is well known from orthopaedic textbooks but its quantitative basis is weak. In order to test the possible results of the principle we performed a study on the effect of subluxation on bone growth and joint deformation in shoulders of children with an Obstetric Brachial Plexus Lesion (OBPL), a lower motor neuron disorder. This neural lesion often results in dorsal humeral head subluxation (subsequently referred to as subluxation) and deformation of the shoulder [9]. In the normal shoulder the glenoid is in mild retroversion with consequently the dorsal length of the scapula being slightly shorter than the ventral side. When the shoulder subluxates retroversion increases and deformation of the normal flat/concave glenoid develops [9].

Based on the Hueter-Volkmann law, we stated the hypothesis that with increasing dorsal subluxation of the humeral head and thus presumably more pressure on the dorsal side and less pressure on the ventral side, the growth of the dorsal length of the scapula would decrease and that the growth of ventral length of the scapula would increase.

## Methods

### Patients

**Figure 1:**
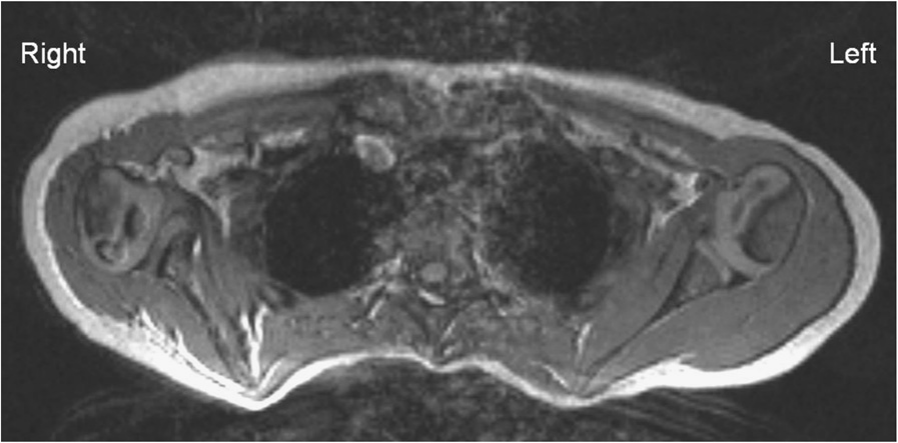
**Transversal MRI projection.** FISP acquisition MRI in axial plane showing affected and normal contralateral shoulder. In the affected right shoulder there is a convex glenoid form (Birch type 2) and humeral head subluxation. The contralateral left shoulder is normal.

In this retrospective study, 58 infants and children with a mean age of 19.7 months (range 1.1 -88.1 months) were included, of which were 29 boys and 29 girls. The study was in accordance with the rules of the Medical Ethical Examination Committee of the VUmc. Children were included if they had unilateral OBPL Narakas classes I to III (I: C5-6, II: C5-6-7, III: C5-6-7-8 [10]) and a good quality MRI of the shoulders was available. MRI were made during neurosurgical analysis or because of the presence of secondary deformities. Posture was standardized with patients in supine position with elbows on the table and flexed 90°, with both hands on their abdomen. In children younger than 4 years MRI was performed under general sedation according to our protocol. The shoulders were visualized with a three-dimensional fast imaging with steady-state precession pulse-acquisition sequence imager (TR 25 msec, TE 10 msec, flip angle 40°). The partitions we used ranged from 0.8 to 1.6 mm. The protocol included transversal projections of both the affected and normal shoulders to enable comparison with the normal anatomy (Figure 1). Software from Centricity RA 600 (General Electric health care, Slough, United Kingdom) was used to take measurements on the MR images.

### Scapular growth, subluxation and joint deformation

**Figure 2:**
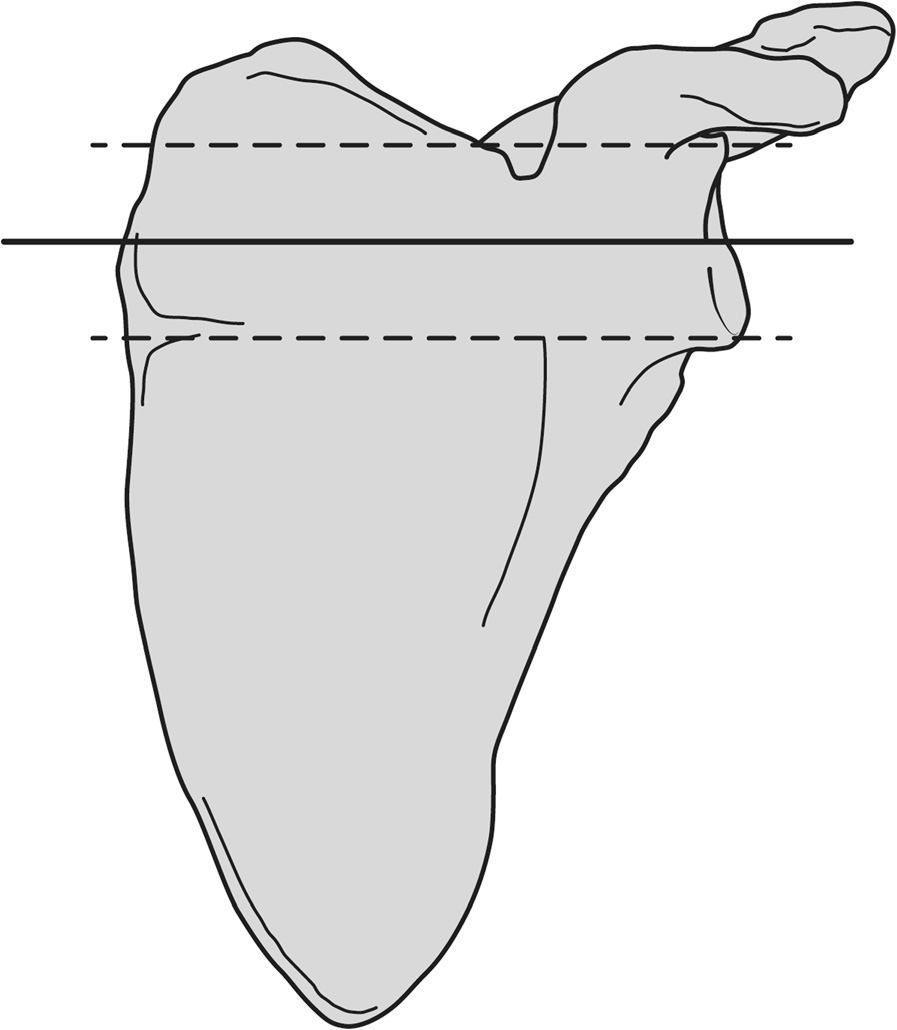
**Mid glenoid level.** On MRI partitions in axial plane the upper and lower end of the glenoid were defined (broken lines). The level exactly between these ends (solid line) was determined to be the mid glenoid level, at which measurements were done.

**Figure 3:**
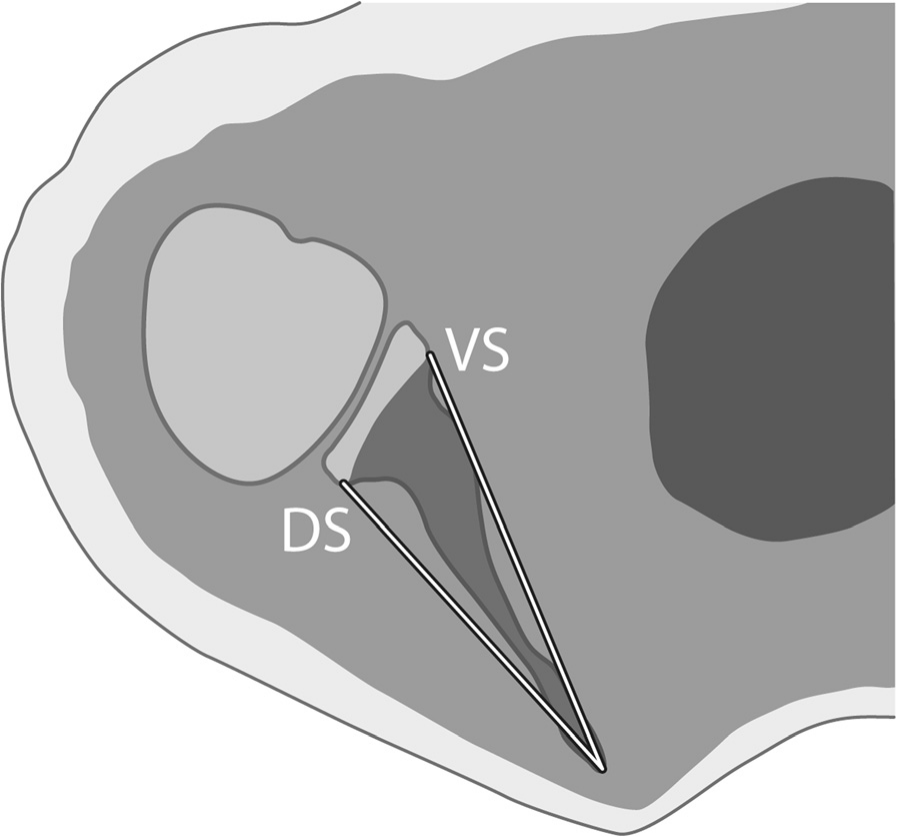
**Method of measuring scapula lengths.** Schematic drawing showing the method of measuring the lengths of ventral (VS) and dorsal(DS) side off the scapula at mid glenoid level.

**Figure 4:**
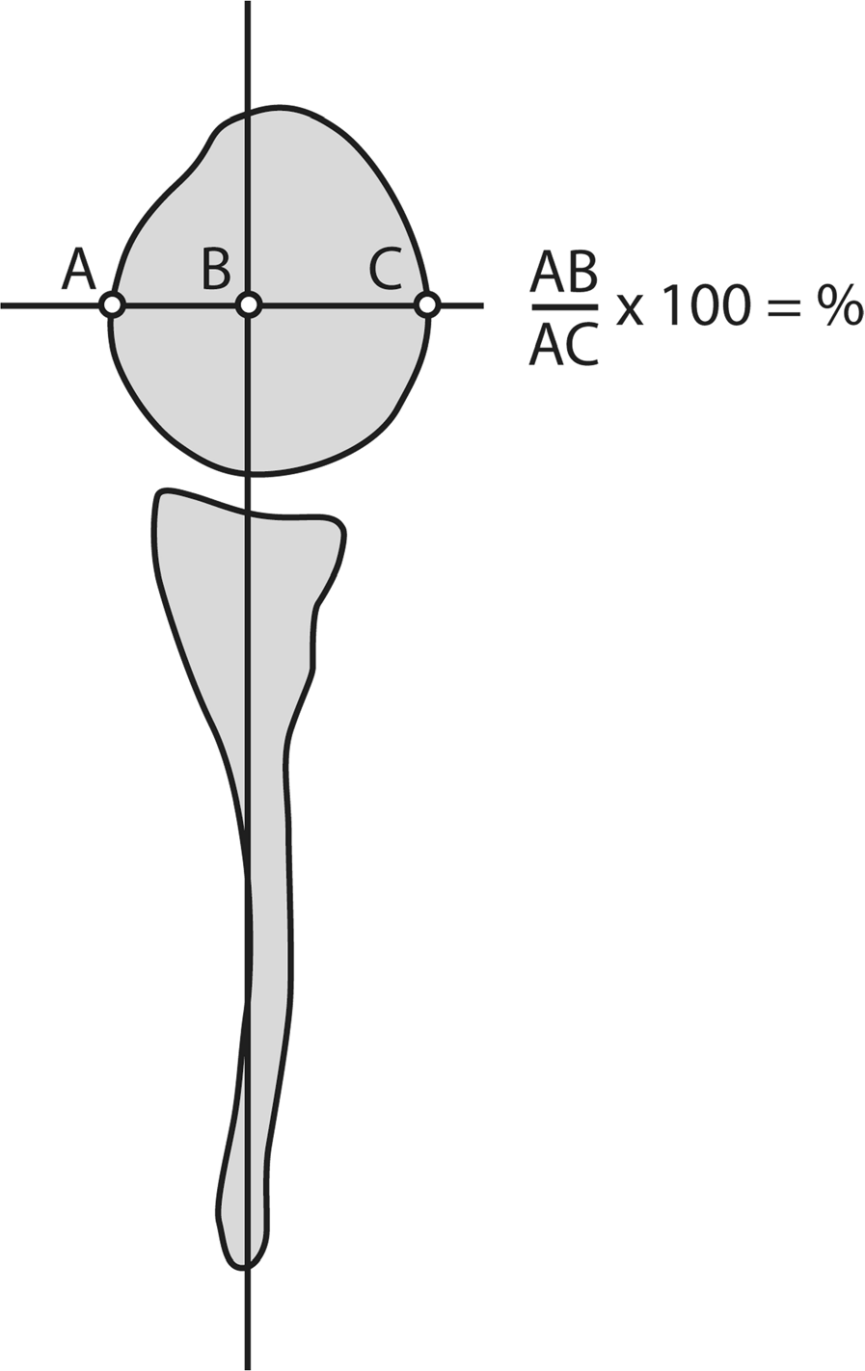
**Method of measuring subluxation.** Schematic drawing showing the method of measuring humeral head subluxation. The percentage of the humeral head anterior to the drawn line indicates subluxation.

All measurements were made on both normal and affected shoulders to enable comparison, assuming that aberrant measurements on the affected side (compared to the normal side) are caused by altered bone growth. For scapular dimensions we measured at mid glenoid level (Figure 2) the horizontal distance between the medial side of the scapula and the subchondral glenoid. This was done for both the ventral side of the scapula (VS) and dorsal side of the scapula (DS) (Figure 3). The ratio between VS and DS (VS/DS) was calculated to assess scapular deformity. Dorsal subluxation of the humeral head was measured quantitatively according to Waters et al. [11]. For this method a line from the medial margin of the scapula through the mid point of the glenoid is drawn through the humeral head (Figure 4). Subluxation is expressed as the percentage of the part of the humeral head anterior to this line. The normal value for this parameter is approximately 50%. In dorsal subluxation, this value decreases. As a qualitative measure of the glenoid form we used the system proposed by Birch, et al. [12] class 1: concave-flat, class 2: convex and class 3: biconcave.

### Statistics

Results are presented as mean (SD). Age and inter-individual differences were corrected by expressing scapular length of the affected side as a ratio of the normal side. Data were analysed using SPSS (version 15.0). Differences were tested using t-test and intra-variance measures with ANOVA. The relation between growth and subluxation was analysed using regression analysis. P < 0.05 was considered to be significant and all analyses were two-tailed.

## Results

In total 58 children with unilateral OBPL were analysed. In 25 children the left side was affected, in 33 the right side. Narakas classes were: class I, 34 children; class II, 12 children and class III, 12 children. There were no complications related to the MR imaging protocol.

### Normal scapula

In the normal scapula ventral length (VS) is longer than dorsal length (DS) (p < 0.001, Table 1) and both are longer than on the affected side. VS and DS on the normal side are related to age (R^2^ =0.7, p < 0.001, Table 2).

**Table 1:** Values of scapular length on ventral and dorsal side of affected and normal shoulder

		**Min (mm)**	**Max (mm)**	**Mean (mm)**	**Std.Deviation (mm)**
Affected side	Ventral	21,2	62,5	39,2* **	9,5
	Dorsal	17,8	56,1	35,8*	8,1
Normal side	Ventral	23,3	72,1	43,6**	9,2
	Dorsal	22,5	70,8	42,3	9,3

* difference compared to normal side p < 0.001.

** difference compared to dorsal side p < 0.001.

**Table 2:** Regression models for scapular length on ventral and dorsal side of normal and affected shoulder related to age (months), all significantly related to age (p < 0.001)

		**Intercept (mm)**	**coefficient**	**R2**
Affected side	Ventral	32.7	0.33	0.59
	Dorsal	30.7	0.26	0.5
Normal side	Ventral	36.9	0.34	0.69
	Dorsal	35.4	0.35	0.7

### Affected scapula

**Figure 5:**
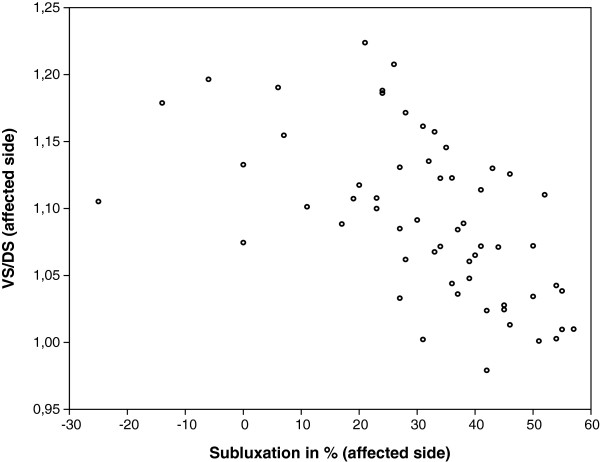
**Subluxation and VS/DS.** Relation between joint subluxation and ratio of scapular length on ventral and dorsal aspect (VS/DS) in the affected shoulder. Increasing posterior subluxation (normal value is 50%, with increasing subluxation this percentage decreases) leads to predominant reduction of scapular length on the posterior side, which leads to an increased ratio VS/DS.

Compared to the normal side, in the affected scapula both VS length and DS length were shorter (p < 0.001, Table 1), resulting in an overall smaller scapula. The reduction of growth on the affected side was more marked on the dorsal side than on the ventral side: mean 6.5(6.7) respectively 4.5(6.6) mm, (p < 0.001, Table 1), corresponding to a 15% (DS) respectively 10% (VS) smaller scapular length. Length on the ventral and dorsal side was related to age (R^2^ = 0.6 respectively 0.5, p < 0.001, Table 2). Analysed separately, the reduction of growth expressed as ratio affected /normal side of either ventral or dorsal scapular length was not related to humeral subluxation (R^2^ = 0.00, p = 0.9 resp R^2^ = 0.03, p = 0.2). However, the form and orientation of the glenoid was determined by the combined effect of ventral and dorsal growth and can be expressed as the ratio of ventral and dorsal scapular length side (VS/DS). Scapular shape, as expressed by the VS/DS ratio was 1.03 (0.05) on the normal side. The VS/DS ratio of the affected side was 1.09 (0.06) (p < 0.001). On the affected side, there was a strong relation with humeral subluxation (R^2^ = 0.33, p < 0.001, Figure 5). With increasing subluxation the VS/DS balance was shifted to dorsal growth reduction (Figure 5). This ratio was also related to age (R^2^ = 0.19, p < 0.001).

Qualitative measurements, as defined by Birch, showed increasing deformation related to changes in the VS/DS ratio. A biconcave glenoid (Birch class 3) was related to more severe dorsal growth reduction. Mean VS/DS ratio in class 1: 1.05 (0.05), in class 2: 1.07(0.05) and in class 3: 1.15(0.05) (p < 0.001).

## Discussion

This study is the first attempt to qualify the possible effects of the Hueter-Volkmann law in the immature shoulder joints. We tried to demonstrate and to qualify the Hueter-Volkmann law (stating that an increase of pressure leads to reduction of bone growth and decrease of pressure leads to stimulation of bone growth) in the development of scapular deformity in children with dorsal subluxation of the humeral head caused by OPBL. We assumed that dorsal subluxation of the humeral head in OPBL leads to increase of pressure at the dorsal side of the glenoid and decrease of pressure at the ventral side of the glenoid. According to the Hueter-Volkmann law, we hypothesized that this aberrant pressure would lead to decreased growth of the dorsal scapula and increased growth of the ventral scapula. However, in contrast to our hypothesis, we could not fully confirm this law in this study of shoulder deformities in children with OPBL. We showed that growth reduction was present both at the ventral and dorsal side of the scapula, with most of the reduction on the dorsal side. On the ventral side of the scapula, where glenohumeral pressure is reduced, there was no increase in growth.

We used a clinical neuromuscular model of subluxation of the shoulder caused by OPBL to qualify the Hueter-Volkmann law. However, we could not prove the Hueter-Volkmann law exactly in our neuromuscular model. It may be questioned why the Hueter-Volkmann law does not fully apply for children with subluxation resulting from OPBL. Hueter describes in his original study the idea that movements and forces are factors influencing joint form and illustrates this on the knee joint [1]. In an experimental setting, growth plates of various animals in various locations show the same reaction to altered mechanical compression and distraction forces [13]. Scapular growth is to some extent different from growth of long bones. It resembles growth of the acetabulum and iliac bone. In the scapula, two growth plates are present: a growth plate along the margo medialis and a growth plate at the glenoid [14]. These two growth plates contribute both to scapular growth but their relative contributions are unknown. This influences the coefficients in the equations on scapular length. It seems intuitively correct to assume that subluxation causes growth reduction primarily on the glenoid growth plate. This means that the coefficients in the equations are larger than given: if the glenoid side contributes 50% of total growth it would result in doubling of the coefficients and even stronger evidence of our findings. Moreover, forces in a neuromuscular disorder differ from physiological forces acting on the joints of long bones. Not only the direction but the nature of forces active is relevant: static compression decreases biosynthetic activity [15], whereas cyclic hydrostatic pressure increases activity [16]. The combined effect of subluxation and abnormal movement caused by neuromuscular dysfunction results in reduced cyclic loading and more static compression on the glenoid, both leading to a reduced local growth and subsequent joint deformation.

Contrary to the Hueter-Volkmann law stating that a reduction of pressure leads to stimulation, we could not find a stimulating effect on growth of the ventral side following reduced ventral “pressure” in case of dorsal subluxation. Growth reduction is present on both the ventral and dorsal side of the scapula, but differs with most of the reduction on the dorsal side. This asymmetry in reduction affects joint development and leads to glenoid and humeral head deformation [9]. Glenoid form and orientation are determined by the combined growth of ventral and dorsal length. Subluxation of the humeral head leads to changes in this balance, leading to increased retroversion or glenoid deformation. In addition we showed that the VS/DS ratio, the balance between ventral and dorsal length of the scapula, is strongly related to the extent of joint subluxation (R^2^ = 0.33, p < 0.001).

The relation between stress and growth is complex: as mentioned in the introduction an increase in stress would first lead to increased growth and excessive stress would lead to retardation [5-8]. However, with no or very little stress growth proceeds at a basal rate [5,7], which we think is the fact on the ventral side of the by OBPL affected scapula. In the normal scapula optimal pressure at both ventral and dorsal side results in a higher (and physiological) growth rate, resulting in optimal and symmetrical lengthening. On the dorsal side of the affected scapula excessive stress of the subluxated humeral head leads to retardation of growth, to the extend that it comes under the basal growth rate at the ventral side. To summarize: growth rate on ventral and dorsal side are reduced because of the paresis in OBPL, on the ventral side because of stress reduction, while on the dorsal side growth is even more severely reduced because of increased stress.

In an effort to demonstrate and qualify the Hueter-Volkmann law in children with OPBL, we encountered some strong points and some weak points. Strong points of our study are the homogeneous set of infants and children, the imaging technique suited for cartilaginous structures and for contralateral comparison. We did not measure growth intra-individually over time, so growth was estimated by the inter-individual differences in age. All measurements and ratio-calculations were intra-individually as we could compare affected scapulas to the normal contralateral scapulas. However, our study has some limitations. One limitation is that scapular growth is different from growth of long bones, as stated earlier. As explained, the presence of two growth plates could theoretically make our findings stronger. Another limitation is that measurements were done on transversal MRI projections that were standard used for imaging of shoulder deformity in OBPL [9,11]. However in OBPL both size and tilt of the affected scapula are different and this affects length measurements [17]. The affected scapula is tilted in several planes. In the coronal plane the inferior angle of the scapula is tilted medially-upward [17]. This tilt leads to a systematic overestimation of the lengths measured on the affected side. This means that growth reduction is larger than described in this study and our findings would even be stronger.

## Conclusion

In conclusion: the paraphrased Hueter-Volkmann law applies partially for children with dorsal subluxated shoulders in OBPL. Dorsal subluxation in OBPL shoulders is associated with decrease of growth of both the dorsal and ventral length of the scapula. Decreased stress did not lead to increased growth of the ventral length of the scapula. According to this study the Hueter-Volkmann law for the subluxated shoulders should be: excessive pressure to a part of the joint leads to more growth retardation of that part of the joint and to less growth retardation of the not stressed part of the joint.

## Abbreviations

OBPL: Obstetric brachial plexus lesion; Subluxation: Dorsal subluxation of the humeral head; VS: Distance between the medial side of the scapula and the subchondral glenoid at the ventral side of the scapula; DS: Distance between the medial side of the scapula and the subchondral glenoid at the dorsal side of the scapula.

## Competing interests

The authors declare that they have no competing interests.

## Authors’ contributions

VMVG: study design, data acquisition, analysis, interpretation and writing. BJR: interpretation of data and critical revision of the manuscript for important intellectual content. JAS: study design, interpretation of data and writing. All authors read and approved the final manuscript.

## Pre-publication history

The pre-publication history for this paper can be accessed here:

http://www.biomedcentral.com/1471-2474/14/107/prepub
